# Investigating the Early Stages of Person Perception: The Asymmetry of Social Categorization by Sex vs. Age

**DOI:** 10.1371/journal.pone.0084677

**Published:** 2014-01-21

**Authors:** Jasmin Cloutier, Jonathan B. Freeman, Nalini Ambady

**Affiliations:** 1 Department of Psychology, University of Chicago, Chicago, Illinois, United States of America; 2 Dartmouth College, Hanover, New Hampshire, United States of America; 3 Stanford University, Palo Alto, California, United States of America; CSIC-Univ Miguel Hernandez, Spain

## Abstract

Early perceptual operations are central components of the dynamics of social categorization. The wealth of information provided by facial cues presents challenges to our understanding of these early stages of person perception. The current study aimed to uncover the dynamics of processing multiply categorizable faces, notably as a function of their gender and age. Using a modified four-choice version of a mouse-tracking paradigm (which assesses the relative dominance of two categorical dimensions), the relative influence that sex and age have on each other during categorization of infant, younger adult, and older adult faces was investigated. Results of these experiments demonstrate that when sex and age dimensions are simultaneously categorized, only for infant faces does age influence sex categorization. In contrast, the sex of both young and older adults was shown to influence age categorization. The functional implications of these findings are discussed in light of previous person perception research.

## Introduction

The ease with which we can infer a wealth of social information from newly encountered individuals has been demonstrated across a range of social cognitive investigations. Amongst the processes utilized to make sense of our social environment, social categorization is believed to be one of the most prevalent and efficient [1]–[4]. Indeed, access to major social categories such as the sex, age and race of social targets has repeatedly been found to dominate the early stages of person perception and to have important downstream consequences for how we construe others (i.e. stereotyping and prejudice) [5]–[7].

More recently, a focus on how such information is extracted from the faces of social targets has confirmed the importance of early perceptual operations in the processing stream leading to social categorization, stereotyping and, more broadly, impression formation [8]–[14]. For instance, certain social category memberships may interact with one another either because the facial cues supporting those memberships are shared, the conceptual knowledge (stereotypes) tied to those membership are shared, or both; such category interactions are accounted for my recent models of person perception [15]–[16]. For example, facial displays of emotion interact with race category, race category interacts with sex category, and many other interactions may occur when perceiving others [17]–[21].

In this burgeoning area of recent work examining social category interactions, age has received considerably less attention than other major category dimensions such as sex or race. The dynamics underlying sex categorization from faces, and its relationship with identity processing, have been extensively investigated from both behavioral and brain imaging perspectives [16] [22]–[25]. Although facilitated by the availability of sex-stereotyped cues [26], even infants have been shown to be able to categorize faces based on sex [27]–[28]. In contrast, even if considered to be a fundamental social category guiding how we perceive others [4] [29]–[30], age has traditionally been understudied in the context of person perception [31]. Notwithstanding the relative lack on research on the topic, evidence suggests age often may take precedence over the sex of social targets during person perception [32]–[33] and has been found to influence the sex categorization of younger and older adults [34]. Furthermore, using repetition priming, a recent study found that sex categorization of younger and older adults may spontaneously occur during age judgments but not vice-versa [35], whereas a previous study found repetition priming effects only for faces encoded on the same dimension, i.e., either sex or age [34]. Despite advances towards understanding how these two fundamental social categories interact during person perception, methodological differences across previous studies [16] and the absence of investigations including younger social targets (i.e. infants) highlight the need for further investigations on the dynamics of age and sex categorization from faces.

Using a computer mouse-tracking paradigm sensitive to the relative dominance of two categorical dimensions [36], the current study aims to identify the relative influence that the dimensions of sex and age may have on each other during social categorization. One possibility is that sex and age are processed independently, which has often been implicitly assumed in social cognitive models of person perception that do not focus on the categorization process e.g. [3]–[4]. Another possibility is that sex and age may exert mutual influences on one another during person perception, even when irrelevant to the processing goal of the perceivers. Such a premise is consistent with recent connectionist models of person perception [15] see also [37]. Indeed, research finding that multiple social dimensions mutually influence one another during the categorization process [12] [15] [20] [36] [38] suggests that such interactions may indeed occur. Functionally, the interaction of these two category dimensions may be dependent on the specific memberships involved. That is, for adult perceivers, the functional significance of gender may be high for other adult faces but arguably quite low for infants, because the sex of similarly aged targets is most motivationally relevant for adult perceivers. Thus, from this perspective [31] [39], sex category—even when not focal for the task—may influence age categorization only when functionally relevant, i.e., for adult faces. In the present work, we examined the relative influences of sex and age categories on the real-time social categorization process.

### Study 1A and 1B

Perceivers were asked to categorize the age and sex of older adult and younger adult faces (Study 1A) or to categorize the age and sex of infant and younger adult faces (Study 1B). The mouse-tracking paradigm allowed us to identify the relative activation of the task-irrelevant category dimension when perceivers were presented with targets from varying age groups (i.e., age during sex categorization and sex during age categorization); [36]. As such, the unintended influence of one dimension over the other may reflect the relative functional significance of sex and age categories when perceiving faces of these three different age groups.

## Methods

### Participants

20 undergraduates participated in Study 1A (*Perceiving young and older adults*), 4 of which were excluded for not following instructions, and 21 participated in Study 1B (*Perceiving younger adults and infants*) for partial course credit or $10. All research was conducted in accordance with the ethical guidelines of the Tufts University IRB. Approval was obtained by the Tufts University IRB and participants provide their written informed consent to participate in the study. No minors/children participated in the study.

### Stimuli

Images of 90 unfamiliar individuals’ faces were obtained from public domain websites. Based on a pilot study with 9 participants, faces from each group did not differ in eye or head gaze, were free of glasses, and had identifiable gender. All images were grayscaled and standardized to a width of 200 pixels and a height varying between 200–300 pixels. Of the 90 images, 30 depicted younger adults, 30 depicted older adults, and 30 depicted infants (with 15 men and 15 women in each age group). Although the exact age for the infant faces was not available, the faces were selected to appear, on average, to be about 2 years old (faces appearing to be of much younger infants were not selected).

A separate group of 20 raters [*M*_age_ = 37.80 (*SD*_age_ = 16.45); 9 females, 6 males; technical issues prevented us from collecting demographic data from 5 participants] provided judgments of the perceived age (i.e., please provide an estimate of the age of these faces) and typicality (i.e., how typical of a male/female younger adult/older adult is this face?) of each face. Typicality judgments were performed on a scale from 1 (not typical at all) to 7 (very typical). On average, older adult faces were perceived to be 71.16 years old and to be typical of their category [older male faces: *M*_age_ = 69.23 (*SD*_age_ = 4.85) and typicality, *M*_age_ = 5.94 (*SD*_age_ = 0.30); older female faces: mean age, *M*_age_ = 73.09 (*SD*_age_ = 7.79) and typicality, *M*_age_ = 5.76 (*SD*_age_ = 0.47). On average, younger adult faces were perceived to be 26.74 years old and were also judged to be typical of their category [younger male faces: mean age, *M*_age_ = 27.72 (*SD*_age_ = 6.29) and typicality, *M*_age_ = 5.49 (*SD*_age_ = 0.29); younger female faces: mean age, *M*_age_ = 25.75 (*SD*_age_ = 2.47) and typicality, *M*_age_ = 5.76 (*SD*_age_ = 0.25). On average, infant faces were perceived to be 3.60 years old and were also judged to be typical of their category [infant male faces: mean age, *M*_age_ = 3.38 (*SD*_age_ = 1.89) and typicality, *M*_age_ = 5.92 (*SD*_age_ = 0.35); younger female faces: mean age, *M*_age_ = 3.83 (*SD*_age_ = 1.48) and typicality, *M*_age_ = 5.96 (*SD*_age_ = 0.29). For Study 1A, the images of younger adults and older adults were used (60 total); for Study 1B, the images of younger adults and infants were used (60 total).

#### Procedure

**Figure 1:**
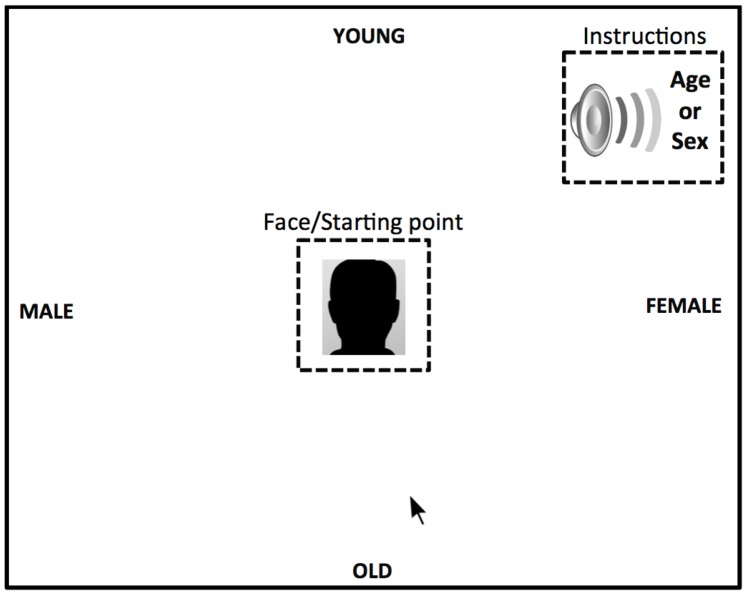
Figure displaying an example of the mouse procedure assessing the relative dominance of two social categories (i.e., sex and age) utilized in study 1a and 1b. During this task participants were given auditory instructions to categorize faces based on either their sex or their age.

Four category responses appeared on the screen: Male, Female, Young, Old. These were equidistant from the center; two labels were directly above/below the center, and two to the left/right of it. The assignment of the categories to the label locations was randomized across participants, but the pair of sex categories and pair of age categories were always located either above/below or left/right (see figure 1).

To start each trial, participants clicked a start button located at the center of the screen. After clicking this, a voice saying “sex” or “age” played, and the target face (either young or older adults for Study 1A, and either younger adults or infants for Study 1B) replaced the start button. The voice was presented nearly simultaneously with the presentation of the face (30 ms following face onset). Participants were instructed to categorize the dimension specified by the voice (sex/age) as quickly and accurately as possible by mouse-clicking the appropriate category response. The face remained on the screen until a response was made. Before the experiment, participants learned the locations of the category labels in a series of practice trials. Each of the 60 faces was presented twice in the experiment, once for sex categorization and once for age categorization (creating a total of 120 trials).

While participants performed the task, we recorded the streaming *x, y* coordinates of the computer mouse (sampling rate ≈70 Hz). To ensure participants’ movement was on-line with the categorization process (rather than off-line once a decision was already finalized), we encouraged participants to move early. Consistent with previous work [40], if participants initiated movement later than 400 ms following face presentation, a message appeared after the trial informing them to start moving earlier even if not yet fully confident of a response. If participants did not respond by 3000 ms following face presentation, a “time out” message appeared and the trial was discarded. To record and analyze mouse-movement data, we used the freely available MouseTracker software package [41].

### Data preprocessing

We use “correct”/“incorrect” to refer to categories along the focal dimension and “relevant distractor”/“irrelevant distractor” to refer to categories along the non-focal dimension. For instance, if instructed to sex-categorize a young woman, Female would be correct, Male would be incorrect, Young would be the relevant distractor, and Old would be the irrelevant distractor.

Trajectories were normalized into 101 time-steps to permit averaging of their full length across multiple trials. For comparison, all trajectories were remapped such that they were directed at the response at the top, with the relevant distractor located at the response location on the right. This was done by inverting trajectories along the x-axis, y-axis, and/or rotating them 90°. To index the hand’s attraction towards the relevant distractor, we computed MD: the largest x-coordinate deviation from an idealized straight-line trajectory between the center start-position and the correct response. Because, after remapping, an idealized response trajectory is a vertical line (*x* = 0), with the relevant distractor located on the right (*x* >0), positive MD indicates attraction toward the relevant distractor, negative MD indicates attraction toward the irrelevant distractor, and values no different than 0 indicate a lack of interference altogether.

## Results

### Study 1A: Perceiving young and older adults

As this four-choice speeded categorization task was difficult, we expected a substantial number of errors. Trials involving categorization errors (15%) were discarded.

#### Initiation and response times

Initiation and response times were submitted to a 2 (judgment type) x 2 (target age) repeated-measures ANOVA. There were no significant main effects of judgment type or target age, or a significant interaction (all *F*s <2.06, all *p*s >.17). Thus, initiation times did not differ when categorizing the age of older-adults (*M* = 173 ms, *SE* = 31 ms) or younger-adults (*M* = 175 ms, *SE* = 33 ms), nor did they differ when categorizing the sex of older-adults (*M* = 168 ms, *SE* = 32 ms) or younger-adult faces (*M* = 170 ms, *SE* = 34 ms), *t*(15) = 0.29, *p* = 0.78. This ensures that all conditions were similarly on-line with the category selection process.

#### Mouse trajectories

**Figure 2:**
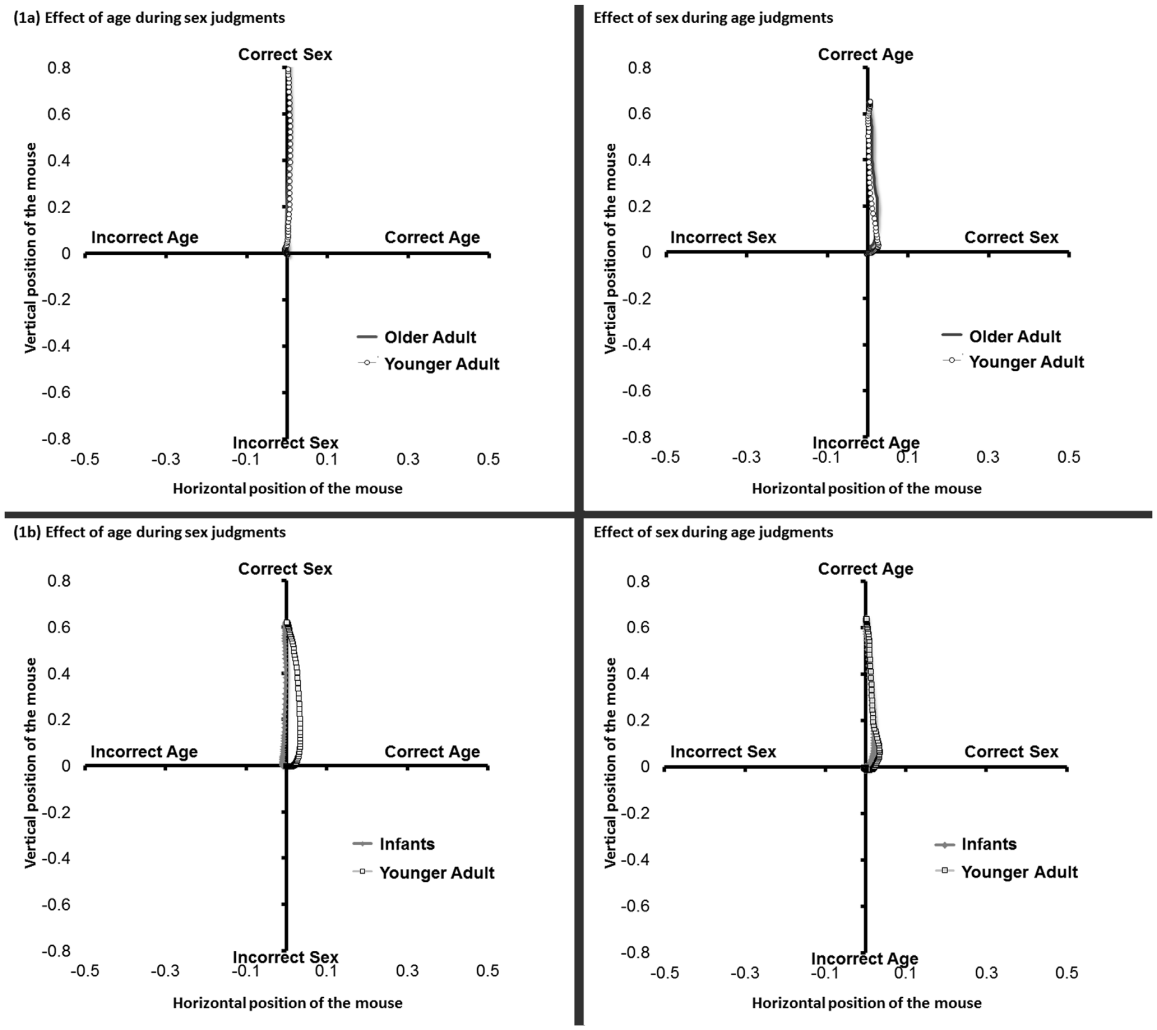
Top: Graphical display of the computer mouse trajectories when categorizing older adults and younger adults faces as a functions of either their sex (left) or their age (right). Inspection of these trajectories reveals: 1) no influence of the relevant age category during sex categorization of both older and younger adult faces; 2) the influence of the relevant sex category during age categorization. Bottom: Graphical display of the computer mouse trajectories when categorizing infant and younger adults faces as a functions of either their sex (left) or their age (right). Inspection of these trajectories reveals: 1) the influence of the relevant age category during sex categorization of infant but not younger adult faces; 2) the influence of the relevant sex category during age categorization of both younger adult and infant faces, with a greater influence when categorizing younger adult faces.

**Figure 3:**
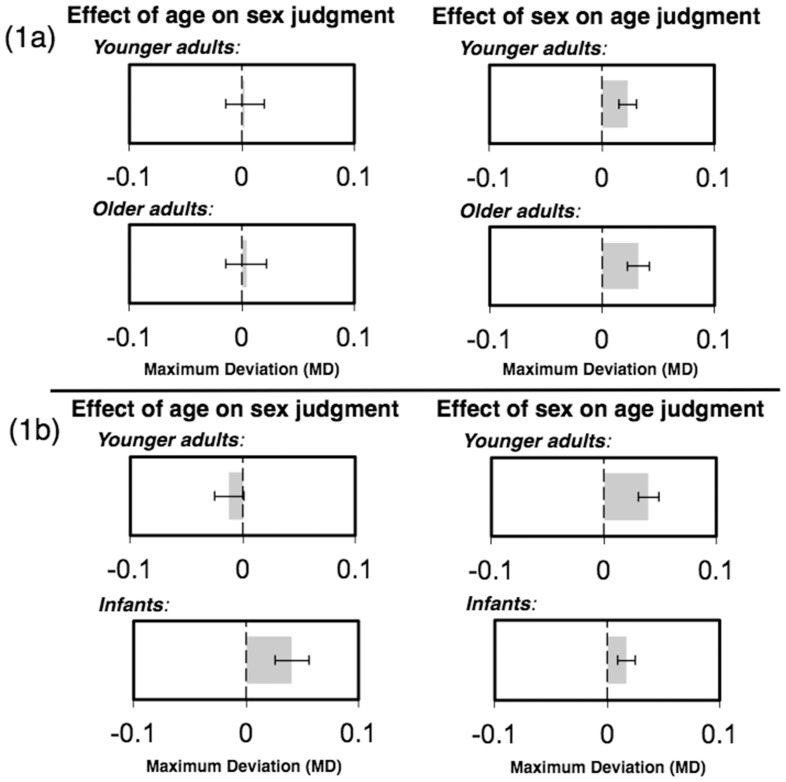
Top: Graphical display of the maximum deviation scores (MD) when categorizing older adults and younger adults faces as a functions of either their sex (left) or their age (right). Bottom: Graphical display of maximum deviation scores (MD) when categorizing infant and younger adults faces as a functions of either their sex (left) or their age (right).

Mouse trajectories for age-categorization trials showed a simultaneous attraction toward the relevant sex category. This was revealed by a significant main effect of judgment type on MD [*F*(1, 15) = 7.32, *p*<.016] with no significant main effect of target age and no significant interaction between judgment type and target age [*F*s<0.19, *p*s>0.66. Further analysis indicated MD (older-adult faces: *M* = 0.032, *SE* = 0.010; younger-adult faces: *M* = 0.023, *SE* = 0.008) being significantly more positive (in the direction of the relevant sex category) than zero for both older-adult faces [*t*(15) = 3.19, *p*<0.006] and younger-adult faces [*t*(15) = 3.06, *p*<0.008], with no significant differences between them: *t*(15) = .84, *p*<0.41 (Figures 2 & 3). Mouse trajectories for sex-categorization trials, however, did not show an attraction toward the relevant age category, with MD (older-adult faces: *M* = 0.004, *SE* = 0.018; younger-adult faces: *M* = 0.002, *SE* = 0.016) not significantly more positive than zero for either older-adult faces [*t*(15) = 0.24, *p*<0.82] or younger-adult faces [*t*(15) = 0.14, *p*<0.89]. There was also no significant difference observed between MD for the older-adult and younger-adult faces: *t*(15) = 0.20, *p*<0.85 (Figures 2 & 3).

### Study 1B: Perceiving younger adults and infants

Trials involving categorization errors (17%) were discarded.

#### Initiation and response time

A 2 (judgment type) x 2 (target age) repeated-measures ANOVA revealed significant effects (all *F*s<0.83, all *p*s >.37). Thus, initiation times did not differ when categorizing the age of infant (*M* = 183 ms, *SE* = 27 ms) and younger-adult faces (*M* = 184 ms, *SE* = 27 ms), *or* when categorizing the sex of infant (*M* = 187 ms, *SE* = 28 ms) and younger-adult faces (*M* = 177 ms, *SE* = 26 ms). As in Study 1A, this ensures that all conditions were similarly on-line with the category selection process.

#### Mouse trajectory

Mouse trajectories for age-categorization trials again showed a simultaneous attraction toward the relevant sex category. This was revealed by a significant main effect of judgment type on MD [*F*(1, 20) = 5.41, *p*<0.031]. Further analysis indicated MD (infant faces: *M* = 0.017, *SE* = 0.008; younger-adult faces: *M* = 0.040, *SE* = 0.009) being significantly more positive (in the direction of the relevant sex category) than zero for both infant faces [*t*(20) = 2.20, *p*<0.039] and younger-adult faces [*t*(20) = 4.35, *p*<0.001], with a significant difference observed between them: *t*(20) = 3.16, *p*<0.005, indicating more attraction towards the relevant sex categories when categorizing younger-adults compared to infant faces (Figures 2 & 3). Mouse trajectories for sex-categorization trials, however, only showed an attraction toward the relevant age category when infant faces were presented. This was indicated by a significant interaction between judgment type and target age on MD [*F*(1, 20) = 10.074, *p*<0.005]. Further analysis found MD for infant faces (*M* = 0.041, *SE* = 0.015) being significantly more positive (in the direction of the relevant age category) than zero, [*t*(20) = 2.68, *p*<0.014] but MD for younger-adult faces (*M* = −0.012, *SE* = 0.013) not being significantly different than zero [*t*(20) = 0.95, *p*<0.36]. A marginally significant difference was observed between the infant and younger-adult faces: *t*(20) = 2.03, *p*<0.056, indicating more attraction towards the relevant age categories when categorizing infant compared to adult faces (Figures 2 & 3). No significant main effect of target age was found [*F*(1, 20) = 1.013, *p*<0.326].

## Discussion

The present work aimed to shed light on the early dynamics of social categorization, focusing on the interplay between the perception of sex and age. The efficiency with which we extract categorical information, such as sex, age, and race, from faces is well documented [10]–[11] [14] [19] [42]. The method utilized in the current study allowed us to extend these findings by identifying the relative impact of sex and age dimensions when both types of social categorization are implemented. Although both are fundamental social categories that often guide person perception [1]–[4], relatively little effort has been devoted to study age categorization or the interaction between age and sex during categorization [31].

The obtained results are not entirely consistent with the results obtained by some previous studies investigating the interaction between sex and age categorization from adult faces [34]–[35]. These differences may be due to the inclusion of faces with a greater age range in the current study (i.e. infant, younger-adult and older-adult faces were included), the different processing operations investigated by the methodologies employed across studies (i.e., mouse-tracking vs. repetition-priming or the Gardner paradigm) or the fact that perceivers in the current study performed, in alternating blocks of trials, both age and sex judgments (i.e. within vs. between-subjects design; [16]. Furthermore, one advantage of the current approach was to identify the relative impact of both dimensions when perceiving faces from distinct age groups (such information is not available when combining age categories into congruent and incongruent priming conditions). Future research is required to further disentangle the early dynamics of person perception across the broad spectrum of social dimensions available from faces (i.e., sex, age, race, and emotion) and at different stages of processing (i.e., initial processing of the faces vs. facilitation or interference effects upon re-encountering social targets).

Our results suggest that, even in early stages of person construal, age may have greater functional significance when perceiving infants. This was demonstrated by an attraction towards the age of infant targets during sex categorization with relatively less attraction towards their sex during age categorization (when compared with the attraction of sex when categorizing the age of younger adult faces). In contrast, when perceiving older-adult faces, like younger adult faces, an attraction was seen towards their sex during age categorization but not vice-versa, suggesting that sex may still have greater functional significance in the early stages of perceiving older adults. This suggests that for younger-adult perceivers, at least in the initial stages of person construal, age is a dominant social category when perceiving infants and sex is a dominant social category when perceiving young and older adults. These findings are broadly consistent with the recently proposed dynamic interactive model of social categorization [15], which argues that bottom-up perceptual cues and top-down social cognitive factors interact may often give rise to various kinds of category interactions (e.g., sex and age). Moreover, a growing body of research has suggested that person perception is inherently combinatorial, in that multiple interesting identities are co-activated and often mutually influence one another [20] [38] [43]. Further experiments independently manipulating bottom-up cues and top-down factors should shed further light on the relative contribution of each in driving interactions of sex and age during early stages of person perception.

The present work also highlights the utility of mouse-tracking for shedding light on social categorization. Mouse-tracking has revealed the real-time dynamics of spoken language processing [44], semantic categorization [45], syntactic ambiguity resolution [46], visual search [47], and attentional control [48]. In the social domain, mouse-tracking has been used to study the real-time dynamics of sex categorization [12], race categorization [49], stereotype activation [50], the explicit reporting of attitudes [51], implicit activation of attitudes [52], and parallel activation of multiple social category dimensions [36]. Here, this technique was able to provide new evidence for the asymmetrical dominance of sex vs. age on the real-time categorization process, in a way that was dependent on the specific memberships associated with each dimension. Thus, future research may wish to use mouse-tracking to provide deeper understandings of a variety of social psychological phenomena.

Functional accounts of face perception may provide insight into the potential communicative value of facial cues across the life span [31] [39]. From this perspective, findings from the current studies may reveal perceptual precursors to the adaptive construal of infant social targets. Indeed, infant faces tend to elicit positive evaluations, as well as strong predispositions to help and act pro-socially [53] [31]. The strength of these predispositions towards infants is further evidenced by the generalization of these responses to adults with “babyfaces” [54]–[55]. The relative dominance of age over sex categorization early in the perceptual process could help initiate and/or contribute to the maintenance of the adaptive construal of infants. In contrast, sex may be the most adaptive social category to guide behavior towards adults, irrespective of their age.

In summary, the current findings suggest that the relationship between distinct perceptually identifiable social dimensions (i.e., sex and age) differs during the categorization of infant, younger adult, and older adult faces. Whereas the sex of both young and older adults influence the categorization of their age, only for infant faces was age found to influence sex categorization. One intriguing possibility is that such dynamics are rooted in the functional characteristics associated with each social dimension across the lifespan [31] [39].
